# Low Prognosis by the POSEIDON Criteria in Women Undergoing Assisted Reproductive Technology: A Multicenter and Multinational Prevalence Study of Over 13,000 Patients

**DOI:** 10.3389/fendo.2021.630550

**Published:** 2021-03-12

**Authors:** Sandro C. Esteves, Hakan Yarali, Lan N. Vuong, José F. Carvalho, İrem Y. Özbek, Mehtap Polat, Ho L. Le, Toan D. Pham, Tuong M. Ho

**Affiliations:** ^1^ ANDROFERT, Andrology and Human Reproduction Clinic, Campinas, Brazil; ^2^ Anatolia IVF, Ankara, Turkey; ^3^ Department of Obstetrics and Gynecology, Hacettepe University, Ankara, Turkey; ^4^ Department of Obstetrics and Gynecology, University of Medicine and Pharmacy at Ho Chi Minh City, Ho Chi Minh City, Vietnam; ^5^ IVFMD, My Duc Hospital, Ho Chi Minh City, Vietnam; ^6^ HOPE Research Center, My Duc Hospital, Ho Chi Minh City, Vietnam; ^7^ Statistika Consulting, Campinas, Brazil

**Keywords:** assisted reproductive technology, POSEIDON criteria, real-world evidence, infertility, prevalence study

## Abstract

**Objective:**

To estimate the prevalence of low-prognosis patients according to the POSEIDON criteria using real-world data.

**Design:**

Multicenter population-based cohort study.

**Settings:**

Fertility clinics in Brazil, Turkey, and Vietnam.

**Patients:**

Infertile women undergoing assisted reproductive technology using standard ovarian stimulation with exogenous gonadotropins.

**Interventions:**

None.

**Main outcome measures:**

Per-period prevalence rates of POSEIDON patients (overall, stratified by POSEIDON groups and by study center) and the effect of covariates on the probability that a patient be classified as “POSEIDON”.

**Results:**

A total of 13,146 patients were included. POSEIDON patients represented 43.0% (95% confidence interval [CI] 42.0–43.7) of the studied population, and the prevalence rates varied across study centers (range: 38.6–55.7%). The overall prevalence rates by POSEIDON groups were 44.2% (group 1; 95% CI 42.6–45.9), 36.1% (group 2; 95% CI 34.6–37.7), 5.2% (group 3; 95% CI 4.5–6.0), and 14.4% (group 4; 95% CI: 13.3–15.6). In general, POSEIDON patients were older, had a higher body mass index (BMI), lower ovarian reserve markers, and a higher frequency of female factor as the primary treatment indication than non-POSEIDON patients. The former required larger doses of gonadotropin for ovarian stimulation, despite achieving a 2.5 times lower number of retrieved oocytes than non-POSEIDON patients. Logistic regression analyses revealed that female age, BMI, ovarian reserve, and a female infertility factor were relevant predictors of the POSEIDON condition.

**Conclusions:**

The estimated prevalence of POSEIDON patients in the general population undergoing ART is significant. These patients differ in clinical characteristics compared with non-POSEIDON patients. The POSEIDON condition is associated with female age, ovarian reserve, BMI, and female infertility. Efforts in terms of diagnosis, counseling, and treatment are needed to reduce the prevalence of low-prognosis patients.

## Introduction

The POSEIDON (**P**atient-**O**riented **S**trategies **E**ncompassing **I**ndividualize**D O**ocyte **N**umber) criteria were developed to help clinicians identify and classify ‘low-prognosis’ patients undergoing assisted reproductive technology (ART) (1, 2). The novel classification aims to capture subtle differences related to a poor treatment outcome, thereby creating homogenous patient groups, ultimately helping clinicians tailor ovarian stimulation strategies for these challenging patients (3).

Since its introduction, the number of clinical studies using the POSEIDON criteria has steadily increased (4–9). However, as yet, there are no global estimates of the real-world prevalence of low-prognosis patients defined according to the POSEIDON criteria.

Prevalence studies assess the burden of a disease or condition in a population and guide clinical practice, research, and resource allocation (10, 11). The accurate interpretation of prevalence studies requires an understanding of the input data on which estimates were based, including quality information, and an explanation of the methods used to derive the health estimates (12).

We investigated the prevalence of POSEIDON low-prognosis patients using big data analytics. Our primary objectives were (i) to determine the prevalence rate of POSEIDON patients in a general infertile population undergoing *in vitro* fertilization/intracytoplasmic sperm injection (IVF/ICSI) and (ii) to identify clinical differences between POSEIDON and non-POSEIDON patients.

## Materials and Methods

### Study Design

This prevalence study is based on retrospective data collected from consecutive infertile patients undergoing IVF-ICSI from October 2015 to November 2017 in three fertility centers (Androfert, Campinas, Brazil, Anatolia IVF and Women’s Health Center, Ankara, Turkey, My Duc Hospital, Ho Chi Minh City, Vietnam). The following ethics committees approved the study: Instituto Investiga, Campinas, Brazil (CAAE 26429219.0.0000.5599), Hacettepe University, Ankara, Turkey (KA-180070), and My Duc Hospital, Ho Chi Minh City, Vietnam (05/18/DD-BVMD). The study complies with the guidelines for accurate and transparent health estimates reporting (GATHER) and standards for the reporting of observational studies (STROBE) (12, 13).

### Study Population

Eligible patients were consecutive infertile women between 22 and 46 years undergoing their first IVF/ICSI cycle in each center with standard ovarian stimulation using exogenous gonadotropins. We included all patients who started treatment regardless of whether their cycle was canceled before oocyte collection. Only one cycle per patient was examined. We excluded patients undergoing IVF/ICSI for purposes other than infertility. We also excluded patients treated with mild or minimal stimulation protocols (i.e., <150 IU daily doses of exogenous gonadotropin, used alone or combined with oral compounds such as anti-estrogens or aromatase inhibitors) and those who underwent natural IVF (*i.e.*, no stimulation) (14). Notably, standard ovarian stimulation is a pre-requisite for classifying a patient according to the POSEIDON criteria (see the *Patient Classification* below).

### Assessment of Ovarian Reserve

The ovarian reserve was determined before and no longer than three months before treatment initiation by measuring either antral follicle count (AFC) or anti-Müllerian hormone (AMH) serum levels, or both, using standardized protocols (15–17). Briefly, the AFC level was measured in the early follicular phase using a two-dimension ultrasound scan (16), whereas AMH serum values were obtained using the modified Beckman Coulter Generation II assay (17). At the ANDROFERT clinic and the My Duc Hospital, both AFC and AMH were routinely used to assess ovarian reserve during the study, whereas at the Anatolia IVF Center, AFC was the preferential method. AFC was determined in-house by the practicing physicians of each study center, whereas AMH values were extracted from reports provided by the reference laboratories partnered with each institution. Thus, AFC and AMH values, critical for the POSEIDON classification (1, 2), were determined by different operators and machines. In the attempt to mitigate this potential source of bias, in the study design phase, we selected study centers that shared similar standard operating procedures for AFC determination and using the same assay for AMH measurements.

### Treatment Protocols

The choice of the ovarian stimulation regimen and gonadotropin dosage was based on each center’s policies according to the ovarian reserve, female age, and history of previous ovarian stimulation (18–22). Patients underwent pituitary suppression with either a long GnRH agonist protocol (Lucrin; Abbott) or a GnRH antagonist protocol (Cetrotide [Merck] or Orgalutran [MSD]). Daily subcutaneous injections of recombinant FSH monotherapy (Gonal-F [Merck] or Puregon [MSD]), recombinant FSH combined with recombinant LH (2:1 ratio, Pergoveris [Merck]), or recombinant FSH (Gonal-F, Merck) combined with either hMG (Menopur, Ferring] or recombinant LH (Luveris, Merck) or highly purified hMG (Menopur; Ferring) were used for ovarian stimulation. The initial daily gonadotropin doses varied between 150 IU and 450 IU.

Ovarian response was monitored primarily using serial transvaginal ultrasonography, and gonadotropin doses were adjusted as needed. Cycles were canceled when no follicles developed during ovarian stimulation. Final oocyte maturation was triggered by subcutaneous administration of either recombinant hCG (250 mcg; Ovitrelle, Merck) or 0.2 mg GnRH agonist (0.2 mg triptorelin [Decapeptyl; Ferring]) according to the policies of each center. Oocytes were retrieved under intravenous anesthesia using transvaginal ultrasound-guided puncture of follicles 35–37 h after triggering final oocyte maturation.

The follicular fluid collected was analyzed in the IVF laboratory, and the total number of retrieved oocytes was recorded. The metaphase II oocytes were inseminated *via* conventional IVF or ICSI, and embryos were cultured up to the cleavage or blastocyst stage. The resulting embryos were either transferred fresh or vitrified according to each center’s policies. In this study, only data up to the number of collected oocytes were considered because this information—in addition to female age and ovarian marker results—is required to classify the patient according to the POSEIDON criteria.

### Data Input

The participating centers used a case report form created for data collection. Data were extracted using each clinic’s data management system: (Clinisys®, Brazil: Androfert; PostgreSQL version 10, USA: My Duc Hospital; a custom-made SPSS-derived database system: Anatolia). Codes replaced the records linking patients’ identification.

The following demographic data were collected: female age, body mass index (BMI), infertility duration, infertility factor, and ovarian reserve markers AFC and/or AMH levels. Treatment data comprised the type of GnRH analog, gonadotropin regimen, total gonadotropin dose, duration of stimulation, and trigger type.

Anonymized individual-level data from the study’s centers were sent to a third-party statistical service (Statistika Co., Campinas, Brazil) for compilation and analysis. Data validation was performed for implausible values due to data entry errors or missing values, and incongruencies were resolved with the principal investigators (SCE, HY and LNV). Individual-level measurements with non-resolved implausible values and/or critical missing values that would preclude the classification of a patient according to the POSEIDON criteria, namely, female age, ovarian biomarker result, and the number of oocytes retrieved (*Patient Classification*) were excluded.

After initial data cleaning, we observed that the number of participants with missing data for AMH was high (**Supplemental Table 1** **)**. While AFC values were reported in virtually all cases by the Brazilian (n = 1,065; 100%), Turkish (n = 3,633; 100%), and Vietnamese (n = 8,448; 92.3%) centers, AMH values were markedly underreported in the Turkish center (n = 425; 11.6%). Therefore, given the aim of the present study, only AFC values were used to classify patients, as detailed in the *Patient Classification* section. We did not generate an indicator based on AFC and AMH when both were available to avoid the risk of having patients without a classification due to a discrepancy between the AFC and AMH results. An agreement analysis between AFC and AMH as a method with which to classify POSEIDON patients does not fall within the scope of the present study; the related data will be reported in a study specifically designed for the matter concerned. Treatment outcomes beyond the number of oocytes retrieved among POSEIDON *versus* non-POSEIDON patients are also beyond this study’s aim and will be reported subsequently. No further data adjustments were made.

### Patient Classification

We classified the patients into five groups based on the POSEIDON criteria (1, 2). Besides the four well-defined POSEIDON groups, patients who did not fit any POSEIDON group were classified into a fifth group designated the “non-POSEIDON” (group 5). The latter group constituted our control group of so-called ‘normal prognosis’ patients.

- POSEIDON Group 1 (Group 1): Age <35 years, an adequate pre-stimulation ovarian reserve biomarker (AFC ≥5), and a previous conventional ovarian stimulation with <10 oocytes retrieved. This group is further divided into subgroup 1a, consisting of patients with fewer than four oocytes and subgroup 1b, consisting of patients with four to nine oocytes retrieved.
- POSEIDON Group 2 (Group 2): Age ≥35 years, an adequate pre-stimulation ovarian reserve biomarker (AFC ≥5), and a previous conventional ovarian stimulation with <10 oocytes retrieved. This group was further divided into subgroup 2a, consisting of patients with fewer than four oocytes and subgroup 2b, consisting of patients with four to nine oocytes retrieved.
- POSEIDON Group 3 (Group 3): Age <35 years and a poor pre-stimulation ovarian reserve biomarker (AFC <5).
- POSEIDON Group 4 (Group 4): Age ≥35 years and a poor pre-stimulation ovarian reserve (AFC <5).
- Non-POSEIDON (Group 5): Patients with an adequate pre-stimulation ovarian reserve biomarker (AFC ≥5) and >9 oocytes retrieved.

### Main Outcome Measures

The primary outcome measure was the prevalence rate of POSEIDON patients (total and stratified by POSEIDON group and by study center) in the dataset (per period prevalence rate). The secondary outcomes were (i) the prevalence ratio of POSEIDON patients among groups and according to study centers, and (ii) the influence of covariates on the probability that a patient be classified as “POSEIDON”. We defined the prevalence rate as the proportion of patients fitting the POSEIDON criteria within the study period. The prevalence ratio was the ratio between the proportion of POSEIDON patients by group and study center.

### Statistical Analysis

The prevalence rates and the simultaneous 95% confidence interval (CI) were computed by the Bonferroni-adjusted method of Goodman (22, 23). The prevalence ratios and associated 95% CI were calculated according to Altman’s method (24). A formal sample size calculation for the estimation of POSEIDON prevalence rates was not carried out *a priori*. However, we included all consecutive patients who met the inclusion criteria and were treated in the study centers over a two-year period. Moreover, we computed CI to determine the statistical precision that was ultimately obtained. The population included in the current study represented 83.6% (13,146/15,728) of all patients treated in these institutions during the same period.

To investigate the relationship between covariates and the condition “POSEIDON”, we performed nominal logistic regression analyses, including the patients’ clinical characteristics (age, infertility duration, AFC, BMI, and infertility factor), and study center as independent variables, and “POSEIDON” [yes = patients fitting the POSEIDON criteria; no = Non-POSEIDON patients (group 5)] as the dependent variable. We explored the above relationships using the POSEIDON patients both as a single category and by subgroup.

For subgroup logistic regression analyses, we combined patients of groups 3 and 4 (poor ovarian reserve) and groups 1 and 2 (sufficient pre-stimulation ovarian reserve and an unexpected poor or suboptimal oocyte yield) into two separate categories. The binary response variables were “POSEIDON groups 3 or 4 = yes; Non-POSEIDON [group 5] = no” and “POSEIDON groups 1 or 2 = yes; Non-POSEIDON [group 5] = no”. We excluded AFC as an independent variable in the model that examined the association between predictors and the condition “POSEIDON groups 3 or 4 = yes” because all patients classified within these groups had low AFC values. We included treatment characteristics (total gonadotropin dose, GnRH analog, type of gonadotropin, duration of ovarian stimulation) as independent variables in the model using “POSEIDON groups 1 or 2 = yes”, because the number of oocytes retrieved—which is a critical variable to classify patients into groups 1 and 2—is affected by treatment. The covariate ‘trigger type’ was excluded from the latter model as the number of POSEIDON patients triggered with a GnRH agonist in the dataset was minimal (217/4,531 = 4.8%; **Supplemental Table 2**).

Categorical data are described by the number of cases, including numerator and denominator, and percentages. Continuous data are reported as median and interquartile range as none of the continuous variables followed a normal distribution. Categorical data were analyzed by Pearson chi-square, whereas continuous data were analyzed by non-parametric tests. The Wilcoxon rank test was used to compare continuous data between POSEIDON and non-POSEIDON patients, whereas the Kruskal-Wallis test was applied for the comparisons among study centers. Statistical significance was set at a p-value <0.05. Computations were carried out using JMP^®^ PRO 13 and SAS 9.3 (SAS Institute, Cary, North Carolina, USA).

## Results

### Participants

**Figure 1:**
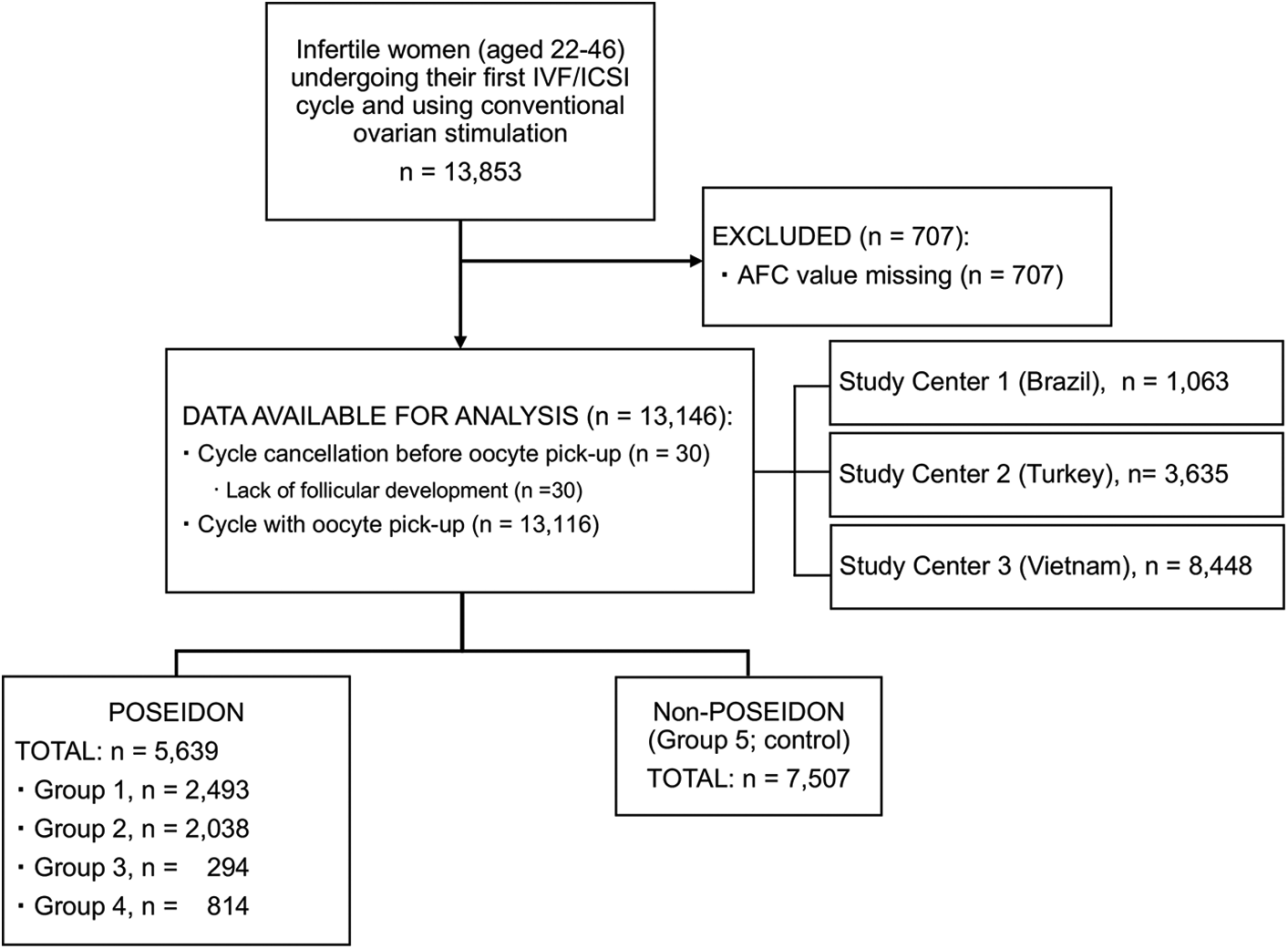
Flow diagram showing total patient breakdown.

Of 13,853 eligible patients, 707 (5.1%) were excluded because AFC values were not reported. Hence, a total of 13,146 patients were included, all of whom had a complete IVF/ICSI record of the relevant covariates for the POSEIDON classification using AFC (**Figure 1**).

**Table 1** shows the study population’s characteristics. A total of 5,639 patients were classified as “POSEIDON”, and 7,507 patients were classified as “non-POSEIDON”. Cycle cancellation before oocyte pick-up was reported in 30 patients (0.23%), all of whom were “POSEIDON” (group 3: n = 14; group 4: n = 16).

**Table 1:** Demographic, clinical, and treatment characteristics of the total studied population, stratified as POSEIDON and Non-POSEIDON patients.

	POSEIDON n = 5,639	Non-POSEIDON n = 7,507	P-value
**Age (years)**	34 [31-38]	31 [28-35]	<0.001^a^
**BMI (kg/m^2^)**	22 [20.0-24.5]	21.3 [19.8-23.7]	<0.001^a^
**Infertility duration (months)**	48 [24-84]	48 [24-72]	<0.001^a^
**Primary indication of ART:**			<0.001^b^
* Male*	1,734/5,639 (30.7)	2,759 /7,507 (36.8)	
* Endometriosis*	763/5,639 (13.6)	279/7,507 (3.7)	
* Ovulatory*	731/5,639 (13.0)	1,105/7,507 (14.7)	
* Tubal*	627/5,639 (11.1)	1,041/7,507 (13.9)	
* Unexplained*	1,784/5,639 (31.6)	2,323/7,507 (30.9)	
**Ovarian reserve:**			
* AFC (n)*	8 [5-12]	17 [13-23]	<0.001^a^
* AMH (ng/mL)*	1.5 [0.87-3.0]	4.9 [3.0-7.8]	<0.001 ^a^
**Duration of stimulation (days)**	9 [8-10]	9 [8-10]	0.48
**GnRH analogue:**			<0.001^b^
* Antagonist*	4,545/5,639 (80.6)	6,629/7,507 (88.3)	
* Agonist*	1,094/5,639 (19.4)	878/7,507 (11.7)	
**Total gonadotropin dose (IU)**	2,700 (1,100-5,100)	2,300 [1,050-4,465]	<0.001^a^
**Gonadotropin:**			<0.001^b^
* HMG*	232/5,639 (4.1)	90 /7,507 (1.2)	
* Rec-FSH*	1,579/5,639 (28.0)	3,431/7,507 (45.7)	
* Rec-FSH+HMG*	3,263/5,639 (57.9)	3,599/7,507 (47.9)	
* Rec-FSH+recLH*	565/5,639 (10.0)	387/7,507 (5.2)	
**Trigger:**			<0.001^b^
* hCG*	5,264/5,639 (93.3)	5,667/7,507 (75.5)	
* GnRH agonist*	375 /5,639 (6.7)	1,840/7,507 (24.5)	
**Number of oocytes retrieved**	6 [4-8]	15 [12-19]	<0.001^a^

^a^Wilcoxon test; values are median and 25%-75% interquartile range.

^b^Pearson χ2 test. Values are number (percentage).

BMI, body mass index; AFC, antral follicle count; AMH, anti-Müllerian hormone; ART, assisted reproductive technology; GnRH, gonadotropin-releasing hormone; IU, international units; HMG, human menopausal gonadotropin; rec-FSH, recombinant follicle-stimulating hormone; rec-LH, recombinant luteinizing hormone; hCG, human chorionic gonadotropin.

Patient and treatment characteristics differed between POSEIDON and non-POSEIDON patients. Overall, POSEIDON patients were older, had a higher BMI, lower AFC, and a higher frequency of female factor as the primary indication for ART than non-POSEIDON patients. Moreover, POSEIDON patients had fewer oocytes retrieved than non-POSEIDON patients, despite using a higher total gonadotropin dose for ovarian stimulation. The GnRH antagonist protocol, an association between rec-FSH and HMG, and hCG trigger was the most commonly used ovarian stimulation regimen in POSEIDON and non-POSEIDON patients. Rec-FSH monotherapy and GnRH agonist trigger were more frequently used in non-POSEIDON patients than in POSEIDON patients (**Table 1**).

### Main Outcome Measures

The prevalence rates of POSEIDON patients, both overall and according to POSEIDON groups and study centers, are reported in **Table 2**. POSEIDON patients represented approximately 43% of the overall studied population. Among POSEIDON patients, groups 1 and 2 (*i.e.*, younger and older patients, respectively, with sufficient pre-stimulation AFC values and unexpected low or suboptimal oocyte yield) had the highest prevalence rates (44.2 and 36.1%, respectively), followed by groups 4 and 3 (14.4 and 5.2%, *i.e.*, older and younger patients, respectively, with low AFC). Notably, most patients of groups 1 and 2 (>80%) had a suboptimal (4–9) oocyte yield (**Supplemental Table 2**).

**Table 2:** Prevalence rates of POSEIDON patients in total population, overall and according to POSEIDON groups and study’s center.

	POSEIDON N (%)	Prevalence Rate	95% Confidence Interval
**Overall Population:**			
**Total**	**5,639/13,146**	**42.9**	**42.0-43.7**
Group 1:	2,493/5,639	44.2	42.6-45.9
Group 2:	2,038/5,639	36.1	34.6-37.7
Group 3	294/5,639	5.2	4.5-6.0
Group 4	814/5,639	14.4	13.3-15.6
**Study Center 1:**			
**Total**	**592/1,063**	**55.7**	**52.7-58.6**
Group 1:	126/592	21.3	17.4-25.8
Group 2:	292/592	49.3	44.2-54.4
Group 3	40/592	6.7	4.6-9.8
Group 4	134/592	22.6	18.6-27.2
**Study Center 2:**			
**Total**	**1,783/3,635**	**49.1**	**47.4-50.7**
Group 1:	1,051/1,783	58.9	56.0-61.8
Group 2:	532/1,783	29.8	27.2-32.6
Group 3:	51/1,783	2.9	2.0-4.0
Group 4:	149/1,783	8.3	6.9-10.1
**Study Center 3:**			
**Total:**	**3,264/8,448**	**38.6**	**37.6-39.7**
Group 1:	1,316/3,264	40.3	38.2-42.5
Group 2:	1,214/3,264	37.2	35.1-39.3
Group 3:	203/3,264	6.2	5.2-7.4
Group 4:	531/3,264	16.3	14.7-17.9
**Prevalence ratio of POSEIDON (total) [95% Confidence Interval] among study centers:** 1.13 [1.07-1.21]^SC1xSC2^ 1.44 [1.35-1.53]^SC1xSC3^ 1.27 [1.21-1.32]^SC2xSC3^			
**Prevalence ratio [95% Confidence Interval] of POSEIDON (by group) among study centers:** **Group 1:** 0.36 (0.31-0.42]^SC1xSC2^ 0.53 [0.45-0.62]^SC1xSC3^ 1.46 [1.38-1.54]^SC2xSC3^ **Group 2:** 1.65 [1.48-1.84]^SC1xSC2^ 1.32 [1.21-1.45]^SC1xSC3^ 0.80 [0.74-0.87]^SC2xSC3^ **Group 3:** 2.36 [1.58-3.53]^SC1xSC2^ 1.09 [0.78-1.50]^SC1xSC3^ 0.46 [0.34-0.62]^SC2xSC3^ **Group 4:** 2.70 [2.19-3.35]^SC1xSC2^ 1.39 [1.18-1.64]^SC1xSC3^ 0.51 [0.43-0.61]^SC2xSC3^			

Study Center (SC) 1: ANDROFERT (Brazil); SC2: Anatolia IVF (Turkey); SC3: My Duc Hospital (Vietnam).

The prevalence ratios of POSEIDON groups at each center and between centers are described in **Table 2**. The risk ratio of reporting a POSEIDON patient in study center 1 (ANDROFERT, Brazil) was 1.1 and 1.4 times higher than that of study center 2 (Anatolia IVF, Turkey) and study center 3 (My Duc Hospital, Vietnam), respectively, and it was about 1.3 times higher in study center 2 than in study center 3.

POSEIDON patients’ characteristics stratified by groups are shown in **Supplemental Tables 3** and **4**. Center 1 POSEIDON patients were the oldest, had the highest frequency of having non-male factor as the primary indication for ART, the poorest ovarian reserve, the longest duration of stimulation, and the fewest number of oocytes retrieved. Study center 2 POSEIDON patients were the youngest, had the highest BMI and frequency of unexplained infertility, exhibited higher AFC, required the lowest total gonadotropin dose for ovarian stimulation, and had the highest number of oocytes retrieved. Lastly, study center 3 POSEIDON patients had the lowest BMI and the highest frequency of female factor as the primary indication for ART (**Supplemental Table 4**).

Patient and treatment characteristics differed among POSEIDON groups. Infertility lasted longer in groups 2 and 4 *versus* groups 1 and 3. Most patients had a female infertility factor as the primary indication for ART; however, this proportion was lower in groups 1 and 2 than groups 3 and 4. In group 1, the frequency of patients with unexplained infertility was higher than in the other groups. As expected, patients of groups 1 and 2 had a higher ovarian reserve and more oocytes retrieved than patients of groups 3 and 4. Overall, the GnRH antagonist protocol, recombinant FSH (alone or combined with HMG), and hCG trigger was the most frequently used ovarian stimulation regimen in POSEIDON patients, albeit practices varied across study centers (**Supplemental Table 3**).

### Logistic Regression Analyses

To assess and quantify the relative importance of each independent variable for the “POSEIDON” condition, we entered our data into the logistic regression software and obtained the values reported in **Table 3** and **Supplemental Tables 5** and **6**.

**Table 3:** Association of patient characteristics and the condition ‘POSEIDON’.

Term (unit)	Estimate	Std Error	P value		Odds ratio*	Lower 95%	Upper 95%
Intercept	0.3970	0.2474	0.1086				
Female age (year)	0.0291	0.0053	<0.0001		1.0279	1.0172	1.0386
BMI (Kg/m2)	0.0364	0.0070	<0.0001		1.0373	1.0231	1.0517
Infertility duration (month)	0.0002	0.0005	0.6245		1.0002	0.9992	1.0013
AFC (n)	-0.1944	0.0045	<0.0001		0.8230	0.8157	0.8304
Primary treatment indication (Female factor)	0.3090	0.0342	<0.0001		1.6376^1^	1.4683	1.8265
Study Center (1-3)	-0.4003	0.1160	0.0006		0.3715^2^	0.2617	0.5273
Study Center (2-3)	0.5944	0.0675	<0.0001		2.1975^3^	1.9427	2.4857
Response: POSEIDON=yes Distribution: binomial Estimation method: nominal logistic Number of Parameters: 7 Whole model effect: ChiSquare=4225.79; p<0.0001				BIC: 11134.3 AICc: 11068.4 RSquare: 0.2667 Area under the curve ROC curve: 0.84 Lack of fit test: 0.85			

Study Center (SC) 1: ANDROFERT (Brazil); SC2: Anatolia IVF (Turkey); SC3: My Duc Hospital (Vietnam).

*Per unit change in regressor (independent variable).

^1^Odds ratio for female factor vs. no female factor (unexplained or male factor).

^2^Odds ratio for Study Center 1 vs. Study Center 2.

^3^Odds ratio for Study Center 2 vs. Study Center 3.

**Table 3** shows the logistic regression values for demographic and clinical parameters using “POSEIDON” as a binary response variable in the whole population. A significant regression equation was found (ChiSquare = 4,225.79, df = 7; p < 0.0001), with an R^2^ of 0.27. Female age, BMI, AFC, and presence of a female infertility factor were significant predictors of the POSEIDON condition. Overall, the probability that a patient was classified “POSEIDON” (versus non-POSEIDON) increased as a function of increased age, increased BMI, decreased AFC values and presence of a female infertility factor. A center effect was relevant, which indicates that the above probability varied across study centers.

A significant regression equation was also found when poor ovarian reserve POSEIDON patients (*i.e.*, groups 3 and 4) were combined (ChiSquare = 1,816.97, df = 6; p < 0.0001), with an R^2^ of 0.45 (**Supplemental Table 5**). Female age, BMI, female infertility factor, and infertility duration were significant predictors of the POSEIDON condition among the expected poor ovarian responders. Accordingly, the probability of classifying a patient as POSEIDON group 3 or 4 (*versus* non-POSEIDON patients) increased with age, infertility duration, and presence of female factor infertility. A center effect was not evident in this model.

The logistic regression values associated with the binary response “POSEIDON groups 1 or 2” (*i.e.*, adequate ovarian reserve but low or suboptimal oocyte number) are shown in **Supplemental Table 6**. A significant regression equation was found (ChiSquare = 3,156.77, df = 13; p < 0.0001), with an R^2^ of 0.23. This model retained the relevant predictors shown in the total population model (**Table 3**) and included the type of GnRH analogue, duration of stimulation, and type of gonadotropin as significant predictors of the POSEIDON condition. Accordingly, older age and higher BMI, lower AFC values, and female factor infertility increased the probability of classifying a patient as groups 1 or 2 POSEIDON. Moreover, shorter stimulation duration, use of the GnRH antagonist protocol, and HMG or rec-FSH+HMG (*versus* rec-FSH alone) for ovarian stimulation was associated with an increased probability of a patient being classified as POSEIDON groups 1 or 2 (*versus* non-POSEIDON counterparts). A center effect was relevant in this model, indicating that the above probability varied across study centers.

## Discussion

### Main Findings

We report POSEIDON patients’ per period prevalence rates using real-world data of three fertility centers in Brazil, Turkey, and Vietnam. Approximately 40% of patients undergoing IVF/ICSI with standard ovarian stimulation were classified as “low-prognosis” according to the POSEIDON criteria. Among them, patients with sufficient pre-stimulation AFC values and an unexpected low or suboptimal oocyte number (groups 1 and 2) constituted about 80% of the POSEIDON individuals while patients with a poor ovarian reserve (groups 3 and 4) comprised the remaining 20%. Despite varying across study centers, the prevalence rates were consistent, thus confirming the perception that the low-prognosis patient accounts for a relevant proportion of individuals undergoing ART. In general, we found that the older the patient population and the lower the ovarian reserve, the higher the proportion of POSEIDON patients.

Clinical and treatment characteristics differed between POSEIDON and non-POSEIDON patients. In general, the former patients were older, slightly heavier, and had a lower ovarian reserve. Moreover, they required larger doses of gonadotropin for ovarian stimulation, which, however, were unable to compensate for the low or suboptimal oocyte yield ultimately obtained. On average, the number of oocytes retrieved was 2.5 times lower in POSEIDON patients than in non-POSEIDON patients. In the former patients, these numbers were twice as high in patients of groups 1 and 2 than in groups 3 and 4. Lastly, a known female infertility factor (*e.g.*, endometriosis) or unexplained infertility was more frequent in POSEIDON patients than in non-POSEIDON counterparts. In general, POSEIDON patients were treated with the GnRH antagonist protocol and a stimulation regimen consisting of recombinant FSH combined with LH-activity provided by hMG. The most common trigger method was the hCG trigger, and most embryo transfers were fresh transfers.

### Interpretation

The main aim of this study was to describe the magnitude and distribution of the so-called “low-prognosis” patient in the routine IVF practice. We determined the number of individuals who had “low-prognosis” as defined by the POSEIDON criteria (1, 2) at a particular time (“per period prevalence”).

The narrow prevalence rate confidence intervals support the certainty of our estimates. However, caution should be exercised in generalizing our results because prevalence rates may be influenced by patient characteristics, clinical practices, and diagnosis criteria (11). Our evaluation relied solely on AFC as the ovarian marker criterion with which to classify the POSEIDON patients. Moreover, the profiles of treated patients and treatment practices varied among centers.

We found that advanced female age, decreased ovarian reserve, increased BMI and the presence of a female infertility factor were the POSEIDON population’s main traits. These predictors were deemed relevant to the “POSEIDON” condition in our logistic regression models. We chose models that introduced all covariates at once because it is biologically plausible that interactions among covariates might better explain the associations ultimately observed (3, 20, 25). We also report the adjusted odds ratio of continuous variables per unit change in regression. This method allows a better understanding of the effect magnitude of explanatory variables on the probability of classifying a patient as POSEIDON. For example, according to the regression coefficients obtained with our models, the probability that an exemplary 33-year-old patient, with a BMI of 23 kg/m^2^, AFC equal to 10 and no female infertility factor be classified “POSEIDON” after a standard ovarian stimulation is 42%. This probability increases to 67% in a patient of similar age with a BMI of 27, AFC of 7, and a female infertility factor.

The POSEIDON patients’ prevalence rates were overall high and varied across study centers, both when the population was analyzed as a whole and after subgrouping. We hypothesize that these findings are mainly related to the characteristics of the treated population. For instance, the median female age was the highest in study center 1, whereas ovarian reserve was the lowest, translating into its highest prevalence rates among the three centers. However, other variables may have influenced the overall and per-center prevalence rates. It is plausible that treatment factors (*e.g.*, gonadotropin total dose and stimulation regimen) and the presence of genetic polymorphisms affecting gonadotropins or their receptors might have accounted for the overall high prevalence of groups 1 and 2 patients. Moreover, we did not investigate smoking habits and socioeconomic factors (*e.g.*, income, education, or occupation). In particular, socioeconomic factors might influence the patient’s decision to seek medical treatment. Also, we did we control for patients’ and doctors’ preferences concerning the gonadotropin regimens used for ovarian stimulation. Besides, it has been suggested that ethnicity might be an independent factor that affects the baseline ovarian reserve (26, 27). However, it remains to be established whether the differences in ovarian reserve observed in POSEIDON patients of different ethnic backgrounds are attributable to genetic, nutrition, infertility causes, or lifestyle factors. Consequently, uncertainty in our estimates may be larger than the statistical uncertainty reflected in confidence intervals and logistic regression models.

Notably, our study concerns a retrospective analysis of a large IVF population dataset; patients were stratified according to the POSEIDON criteria *a posteriori*, that is, after finishing the IVF cycle. Thus, the protocols utilized were based on the prevailing practices during the study period, which have not considered the POSEIDON criteria to guide patient management. In these lines, genetic factors, including polymorphisms affecting endogenous and/or their receptors, may also play a role in ovarian response to exogenous gonadotropin stimulation and ovarian reserve (28). Indeed, hypo-response to gonadotropin therapy is still highly undervalued (29). It is therefore possible that we do not adequately stimulate these patients. However, in routine practice, clinicians only detect the hypo-response during or after treatment, unless there is an evident history of hypo-response in previous cycles. POSEIDON patients, particularly those within groups 1 and 2, may harbor genetic variants potentially contributing to the hypo-response. Genotyping before COS could help better personalize treatment protocols (30), but the frequency and impact of specific genotype profiles on ovarian reserve and reproductive outcomes of POSEIDON patients have not yet been studied. Once these gaps in knowledge are filled, the pharmacogenomic-based COS may become a reality for these patients. Nonetheless, further research is needed to clarify the clinical utility of genotyping in low-prognosis patients.

### Clinical Importance

Estimating the prevalence of low-prognosis patients in real-world settings using unified criteria, such as the POSEIDON classification, has relevant clinical implications. Firstly, it may provide information about the frequency of this condition. Secondly, it may reveal possible causal associations between patient characteristics and the low-prognosis status, with implications for research on infertility etiology, clinical practices, and public health policies.

This study is the first to report global prevalence estimates of POSEIDON patients. Herein we show that “low-prognosis” defined according to the POSEIDON criteria is a common condition, albeit with some geographic variations. Overall, our POSEIDON population was characterized by older patients with a lower ovarian reserve than non-POSEIDON counterparts. Furthermore, we found that, on average, POSEIDON patients had a higher BMI and, more often, a female infertility factor associated with this condition (versus non-POSEIDON). The relationship between female age, ovarian markers and ART reproductive success is well-established (31–33), and BMI has been shown to influence the number of oocytes retrieved and embryos obtained, as well as pregnancy outcomes (34).

The association between increased BMI and the condition ‘POSEIDON’ is a novel and interesting finding of our study. We found that BMI was an independent predictor of ‘low-prognosis’ both in the overall POSEIDON population and its subgroups. The regression coefficients obtained from our models indicated that women with expected low ovarian response (groups 3 and 4) were those mostly affected by increased BMI. However, after adjustment for other relevant covariates, the odds ratios obtained for patients of groups 1–2 and groups 3–4 were essentially the same (**Supplemental Tables 5** and **6**). These findings indicate that BMI has a significant effect, albeit of small magnitude, on the risk of ‘low-prognosis’, which seems to affect POSEIDON subgroups similarly and interact with other risk factors.

Our findings may help decision-makers and practitioners implement measures to mitigate the risk of low-prognosis and optimize reproductive planning. Awareness campaigns highlighting the adverse impact of advanced female age and impaired ovarian reserve on reproductive success (35, 36), the effect of lifestyle changes (37–40), and the role of treatment strategies to improve treatment success could be explored (41–46). Although the impact of the above interventions on POSEIDON prevalence rates remains mostly unknown, our data suggest that the “low-prognosis” burden could be prevented at least partially by treating patients earlier.

To date, only two single-center studies have looked at how often POSEIDON patients account for the overall treated population. In one large retrospective study from China, approximately 20% of IVF cycles fit the POSEIDON criteria (9). However, the authors studied cycles rather than patients, thereby precluding an accurate analysis of the real prevalence rates per treated patient population. In another study, also from China, Li and co-workers reported a POSEIDON prevalence rate of 31.5% over a 3-year period (4), which is consistent with the prevalence rate of the Vietnamese center (38.6%) included in our study. Nevertheless, an in-depth evaluation of the features that characterize POSEIDON and non-POSEIDON patients was not possible as the study by Li et al. lacked a control group.

### Limitations

The AFC was determined by different operators using different machines. Although the reported AFC inter-observer variability is low (47), we cannot exclude that variations in technique and reporting have influenced patient classification. Besides, we did not assess prevalence rates using AMH due to excessive missing data. Different prevalence rates might have been obtained if AMH had been used. Another limitation is that referral for ART treatment may differ among study centers, thus affecting the prevalence rates of POSEIDON patients potentially. Besides medical factors, economic and social factors may impact access to treatment. We were unable to control for this potential source of bias and recognize that POSEIDON prevalence rates might differ, for instance, in studies conducted in countries/regions where IVF is publicly reimbursed or recommended earlier. Lastly, our study included only patients who initiated the treatment cycle.

Notwithstanding the above limitations, we are confident that our analysis of a large patient cohort provides a fair representation of real-world IVF practices and that our methods may guide future data collection. Our sample size is large enough to analyze the effect of candidate factors on the occurrence of “low-prognosis”. However, due to the intrinsic limitations of cohort studies like ours to establish causal relationships, readers should interpret the impact of explanatory variables on the POSEIDON condition as associations rather than causation. Our study should be viewed as contributing to the literature and not as a stand-alone basis for inference and action.

### Future Research

Additional real-world studies and pragmatic trials should be carried out to confirm (or refute) our observations, particularly those concerning the contribution of relevant covariates affecting the probability of the condition “POSEIDON”. Moreover, prospective trials looking into the relationship between low-prognosis and gonadotropin receptor polymorphisms, and lifestyle and treatment regimens to mitigate its occurrence may be prioritized.

## Conclusions

The estimated prevalence of POSEIDON patients in the general population undergoing IVF/ICSI is significant. The ‘low-prognosis’ patient defined according to the POSEIDON criteria has distinct clinical characteristics compared to the non-POSEIDON counterpart. The ‘POSEIDON’ condition is associated with female age, ovarian reserve, BMI, female infertility, and possibly ovarian stimulation regimens. Efforts are needed to reduce the prevalence of ‘low-prognosis’ patients by adequate diagnosis, counseling, and treatment.

## Data Availability Statement

The original contributions presented in the study are included in the article/**Supplementary Material**. Further inquiries can be directed to the corresponding author.

## Ethics Statement

The studies involving human participants were reviewed and approved by the following ethics committees: Instituto Investiga, Campinas, Brazil (CAAE 26429219.0.0000.5599), Hacettepe University, Ankara, Turkey (KA-180070), and My Duc Hospital, Ho Chi Minh City, Vietnam (05/18/DD-BVMD). Written informed consent for participation was not required for this study in accordance with the national legislation and the institutional requirements.

## Author Contributions

SE designed and coordinated the study and helped with the data acquisition, analysis, and interpretation. HY and LV designed the study, helped with the data acquisition, analysis, and interpretation. JC helped in designing the study, carried out the statistical analyses, and helped with the data interpretation. İÖ, MP, HL, TP, and TH participated in the data acquisition and article’s revision for critical intellectual content. All authors contributed to the article and approved the submitted version.

## Funding

This study was supported by an unrestricted investigator-sponsored study grant (MS200059_0013) from Merck, Darmstadt, Germany. The funder had no role in study design, data collection, analysis, decision to publish, or manuscript preparation.

## Conflict of Interest

SE is a co-founder of the POSEIDON group and declares receipt of unrestricted research grants from Merck and lecture fees from Merck and Med.E.A. HY declares receipt of payment for lectures from Merck and Ferring. LV receives speaker fees and conferences from Merck, Merck Sharp and Dohme (MSD) and Ferring and research grants from MSD and Ferring. TH received speaker fees and conferences from Merck, MSD and Ferring. JC is an employee of Statistika Consulting.

The remaining authors declare that the research was conducted in the absence of any commercial or financial relationships that could be construed as a potential conflict of interest.
