# Supplementary material for: Low Prognosis by the POSEIDON Criteria in Women Undergoing Assisted Reproductive Technology: A Multicenter and Multinational Prevalence Study of Over 13,000 Patients
Source: Front Endocrinol (Lausanne). 2021 Mar 12;12:630550. doi: 10.3389/fendo.2021.630550 (PMC8006427; doi:10.3389/fendo.2021.630550)
Supplement: Supplementary file 1 [file DataSheet_1.docx]

**Supplementary Table** **1**. Number of eligible participants (N), participants with missing data according to each variable (N missing), and % missing participants per variable.

|  |  |  |  |
| --- | --- | --- | --- |
| **Variable** | **N** | **N**  **Missing** | **% Missing** |
| Female age | 13853 | 0 | 0% |
| AFC | 13146 | 707 | 5% |
| AMH | 10137 | 3716 | 27% |
| Infertility duration | 12741 | 1112 | 8% |
| BMI | 13377 | 476 | 3% |
| Infertility factor | 13853 | 0 | 0% |
| GnRH analog | 13853 | 0 | 0% |
| Gonadotropin regimen | 13853 | 0 | 0% |
| Trigger type | 13853 | 0 | 0% |
| Stimulation duration | 13430 | 423 | 3% |
| Total gonadotropin dose | 13539 | 314 | 2% |
| No. Oocytes retrieved | 13853 | 0 | 0% |
