# Supplementary material for: Low Prognosis by the POSEIDON Criteria in Women Undergoing Assisted Reproductive Technology: A Multicenter and Multinational Prevalence Study of Over 13,000 Patients
Source: Front Endocrinol (Lausanne). 2021 Mar 12;12:630550. doi: 10.3389/fendo.2021.630550 (PMC8006427; doi:10.3389/fendo.2021.630550)
Supplement: Supplementary file 2 [file DataSheet_2.docx]

|  | **No. patients** | **Prevalence rate in total POSEIDON population (%)** | **Intra-group prevalence rate (%)** |
| --- | --- | --- | --- |
| **Overall POSEIDON population, N=5,639** | | | |
| Group 1:  *1a*  *1b* | 2,493  239  2,254 | 44.2  4.2  40.0 | -  9.5  90.5 |
| Group 2:  *2a*  *2b* | 2,038  315  1,723 | 36.1  5.6  30.5 | -  15.5  84.5 |
| **Study Center 1 POSEIDON population, N=592** | | | |
| Group 1:  *1a*  *1b* | 126  12  114 | 21.3  2.1  19.2 | -  9.5  90.5 |
| Group 2:  *2a*  *2b* | 292  77  215 | 49.3  13.0  36.3 | -  26.4  73.6 |
| **Study Center 2 POSEIDON population, N=1,783** | | | |
| Group 1:  *1a*  *1b* | 1,051  87  964 | 58.9  4.9  54.0 | -  8.3  91.7 |
| Group 2:  *2a*  *2b* | 532  50  482 | 29.8  2.8  27.0 | -  9.4  90.6 |
| **Study Center 3 POSEIDON population, N=3,264** | | | |
| Group 1:  *1a*  *1b* | 1,316  140  1,176 | 40.3  4.3  36.0 | -  10.6  89.4 |
| Group 2:  *2a*  *2b* | 1,214  188  1,026 | 37.2  5.8  31.4 | -  15.5  84.5 |

**Supplemental Table 2.** Prevalence rates of POSEIDON patients classified as subgroups ‘a’ and ‘b’ relative to the total POSEIDON population and within groups, overall and by study’s center.
