# Supplementary material for: Low Prognosis by the POSEIDON Criteria in Women Undergoing Assisted Reproductive Technology: A Multicenter and Multinational Prevalence Study of Over 13,000 Patients
Source: Front Endocrinol (Lausanne). 2021 Mar 12;12:630550. doi: 10.3389/fendo.2021.630550 (PMC8006427; doi:10.3389/fendo.2021.630550)
Supplement: Supplementary file 3 [file DataSheet_3.docx]

**Supplementary Table 3.** Demographic and treatment descriptive characteristics of POSEIDON patients, stratified by group and study’s center.

|  |  | **POSEIDON Groups** | | | |
| --- | --- | --- | --- | --- | --- |
|  | **Total**  **n=5,639** | **Group 1**  **n=2,493** | **Group 2**  **n=2,038** | **Group3**  **n=294** | **Group 4**  **n=814** |
| **Age (years)**  *Study Center 1*  *Study Center 2*  *Study Center 3* | 37 [34-40]  32 [29-36]  35 [32-39] | 31 [28-33]  32 [30-34]  30 [27-32]  31 [29-33] | 38 [36-40]  38 [35-45]  37 [36-40]  38 [36-40] | 32 [30-33]  33 [31-33]  31 [28-32]  32 [30-33] | 39 [37-42]  40 [37-43]  39 [37-41]  39 [37-42] |
| **BMI (kg/m^2^)**  *Study Center 1*  *Study Center 2*  *Study Center 3* | 23.7 [21.8-26.4]  24.8 [22.0-28.4]  20.8 [19.6-22.5] | 21.9 [20.0-24.8]  22.8 [21.3-26.1]  24.6 [22.0-28.3)  20.5 [19.3-22.1] | 22.2 [20.4-24.9]  23.9 [22.0-26.1]  25.4 [22.7-28.9]  21.2 [20.0-22.8] | 20.8 [19.5-23.0]  23.2 [20.7-25.0]  23.0 [21.2-26.5]  20.3 [19.1-21.9] | 21.8 [20.2-23.8]  24.4 [22.2-27.8]  24.5 [19.0-37.8]  21.2 [20.0-22.8] |
| **Infertility duration (months)**  *Study Center 1*  *Study Center 2*  *Study Center 3* | 48 [24-96]  48 [24-84]  48 [24-84] | 48 [24-172]  42 [24-61]  48 [25-72]  48 [24-72] | 60 [24-96]  48 [24-120]  48 [24-108]  60 [24-108] | 48 [24-72]  42 [36-84]  36 [18-72]  48 [24-84] | 60 [24-108]  52 [32-120]  48 [24-116]  60 [24-108] |
| **Primary indication ART (Male factor)**  *Study Center 1*  *Study Center 2*  *Study Center 3* | 91/592 (15.4)  540/1,783 (30.3)  660/3,264 (20.2) | 825/2,493 (33.1)  23/126 (18.2)  384/1,051 (36.5)  418/1,316 (31.7) | 394/2,038 (19.3)  56/292 (19.2)  146/532 (27.5)  192/1,214 (15.8) | 26/294 (8.8)  2/40 (0.5)  4/51 (7.8)  20/203 (9.8) | 51/814 (6.2)  10/134 (7.5)  11/149 (7.4)  30/531 (5.6) |
| **Primary indication ART (Female factor)**  *Study Center 1*  *Study Center 2*  *Study Center 3* | 242/592 (40.9)  621/1,783 (34.8)  2,336/3,264 (71.6) | 1,026/2,493 (41.1)  30/126 (23.8)  260/1,051 (24.7)  736/1,316 (56.0) | 1,286/2,038 (63.1)  167/292 (57.2)  188/532 (35.3)  931/1,214 (76.7) | 251/294 (85.4)  35/40 (82.0)  38/51 (74.5)  178/203 (87.8) | 711/814 (87.3)  95/134 (70.9)  125/149 (83.9)  491/531 (92.5) |
| **Primary indication ART**  **(Unexplained infertility)**  *Study Center 1*  *Study Center 2*  *Study Center 3* | 151/592 (25.5)  622/1,783 (34.9)  268/3,264 (8.2) | 642/2,493 (25.7)  73/126 (58.0)  407/1,051 (38.8)  162/1,316 (12.3) | 358/2,038 (17.6)  69/292 (23.6)  198/532 (17.2)  91/1,214 (7.5) | 22/294 (7.5)  7/40 (17.5)  10/51 (19.7)  5/203 (2.4) | 52/814 (6.4)  29/134 (21.6)  13/149 (8.7)  10/531 (1.9) |
| **AFC (n)**  *Study Center 1*  *Study Center 2*  *Study Center 3* | 6 [4-8]  11 [8-15]  7 [5-11] | 11 [8-15]  7 [6-10]  12 [9-17]  10 [7-14] | 8 [6-10]  7 [6-8]  10 [8-12]  8 [6-10] | 3 [3-4]  3 [2-4]  3 [3-4]  3 [2-4] | 3 [2-4]  3 [2-4]  3 [2-4]  3 [2-4] |
| **AMH (ng/mL)**  *Study Center 1*  *Study Center 2*  *Study Center 3* | 0.9 [0.4-1.8]  1.1 [0.6-2.0]  1.7 [1.0-3.3] | 2.5 [1.3-4.4]  1.5 [0.8-2.3]  1.9 [0.9-3.0]  2.7 [1.4-4.6] | 1.6 [1.0-2.8]  1.2 [0.6-2.0]  1.2 [0.7-1.6]  1.8 [1.1-3.1] | 0.7 [0.3-1.2]  0.4 [0.1-0.7]  0.4 [0.1-0.8]  0.9 [0.5-1.4] | 0.7 [0.3-1.2]  0.3 [0.1-0.7]  0.3 [0.2-0.6]  0.8 [0.4-1.2] |
| **Duration of stimulation (days)**  *Study Center 1*  *Study Center 2*  *Study Center 3* | 10 [10-10]  9 [8-11]  9 [8-10] | 9 [8-10]  10 [10-10]  9 [8-10]  9 [8-10] | 9 [8-10]  10 [10-10]  9 [8-11]  9 [8-10] | 9 [8-11]  10 [10-10]  10 [8-11]  9 [8-11] | 9 [8-11]  10 [10-10]  10 [8-12]  9 [8-11] |
| **GnRH analogue (Antagonist)**  *Study Center 1*  *Study Center 2*  *Study Center 3* | 562/592 (94.9)  677/1,783 (37.9)  3,264/3,264 (100.0) | 1,893/2,493 (75.9)  120/126 (95.0)  457/1,051 (43.5)  1,316/1,316 (100.0) | 1,631/2,038 (80.0)  273/292 (93.5)  144/532 (27.0)  1,214/1,214 (100.0) | 256/294 (87.1)  38/40 (95.0)  15/51 (29.4)  203/203 (100.0) | 731/814 (89.8)  131/134 (97.8)  69/149 (46.3)  531/531 (100.0) |
| **GnRH analogue (Agonist)**  *Study Center 1*  *Study Center 2*  *Study Center 3* | 30/592 (5.1) 1,106/1,783 (62.1)  0/3,264 (0.0) | 600/2,493 (24.1)  6/126 (5.0)  594/1,051 (56.5)  0/1,316 (0.0) | 407/2,038 (20.0)  19/292 (6.5)  388/532 (73.0)  0/1214 (0.0) | 38/294 (12.9)  2/40 (5.0)  36/51 (70.6)  0/203 (0.0) | 82/814 (10.1)  3/134 (2.2)  79/149 (53.7)  0/531 (0.0) |
| **Total gonadotropin dose (IU)**  *Study Center 1*  *Study Center 2*  *Study Center 3* | 3,000 [2,250-3,300]  2,025 [1,500-2,925]  3,000 [2,400-3,450] | 2,400 [1,725-3,000]  2,625 [2,025-3,000]  1,800 [1,350-2,400]  2,775 [2,250-3,450] | 2,902 [2,400-3,450]  3,000 [2,250-4,000]  2,598 [1,800-3,300]  3,000 [2,550-3,450] | 3,000 [2,700-3,937]  3,000 [2,362-3,893]  3,300 [2,400-4,500]  3,075 [2,700-3,900] | 3,075 [2,550-4,050]  3,000 (2,250-3,300]  4,050 [2,400-4,950]  3,075 [2,550-3,900] |
| **Gonadotropin (HMG)**  *Study Center 1*  *Study Center 2*  *Study Center 3* | 1/592 (0.1)  142/1,783 (8.0)  31/3,264 (0.9) | 88/2,493 (3.5)  0/126 (0.0)  82/1,051 (7.8)  6/1,316 (0.4) | 48/2,038 (2.3)  0/292 (0.0)  37/532 (7.0)  11/1,214 (0.9) | 11/294 (3.7)  0/40 (0.0)  5/51 (9.8)  6/203 (3.0) | 28/814 (3.4)  0 (0.0)  20/149 (13.5)  8/531 (1.5) |
| **Gonadotropin (rFSH)**  *Study Center 1*  *Study Center 2*  *Study Center 3* | 297/592 (50.2)  784/1,783 (44.0)  526/3,264 (16.1) | 920/2,493 (36.9)  102/126 (81.0)  595/1,051 (56.6)  223/1,316 (16.9) | 491/2,038 (24.1)  123/292 (42.1)  149/532 (28.0)  219/1,214 (18.0) | 60/294 (20.4)  30/40 (75.0)  8/51 (15.7)  22/203 (10.8) | 111/814 (13.6)  42/134 (31.3)  7/149 (4.7)  62/531 (11.7) |
| **Gonadotropin (rFSH+HMG)**  *Study Center 1*  *Study Center 2*  *Study Center 3* | 0/592 (0.0)  713/1783 (40.0)  2573/3264 (78.9) | 1368/2,493 (54.9)  0/126 (0.0)  316/1,051 (30.1)  1,052/1,316 (80.0) | 1205/2,038 (59.1)  1/292 (0.3)  282/532 (53.0)  922/1,214 (76.0) | 196/294 (66.7)  0/40 (0.0)  28/51 (54.9)  168/203 (82.7) | 544/814 (66.8)  0 (0.0)  113/149 (75.8)  431/531 (81.2) |
| **Gonadotropin (rFSH+rLH)**  *Study Center 1*  *Study Center 2*  *Study Center 3* | 294/592 (49.7)  144/1,783 (8.0)  134/3,264 (4.1) | 117/2,493 (4.7)  24/126 (19.0)  58/1,051 (5.5)  35/1,316 (2.7) | 294/2,038 (14.4)  168/292 (57.6)  64/532 (12.0)  62/1,214 (5.1) | 27/294 (9.2)  10/40 (25.0)  10/51 (19.6)  7/203 (3.5) | 131/814 (16.2)  92/134 (68.7)  9/149 6.0)  30/531 (5.6) |
| **Trigger type (hCG)**  *Study Center 1*  *Study Center 2*  *Study Center 3* | 198/592 (33.4)  1,734/1,783 (97.2)  3,223/3,264 (98.7) | 2,394/2,493 (96.0)  91/126 (72.0)  1,020/1,051 (97.0)  1,283/1,316 (97.5) | 1,920/2,038 (94.2)  194/292 (66.4)  520/532 (97.7)  1,206/1,214 (99.3) | 281/294 (95.6)  27/40 (67.5)  51/51 (100.0)  203/203 (100.0) | 616/814 (75.7)  82/134 (61.2)  3/149 (3.0)  531/531 (100.0) |
| **Trigger type (GnRH agonist)**  *Study Center 1*  *Study Center 2*  *Study Center 3* | 394/592 (66.6)  49/1,783 (2.8)  41/3,264 (1.2) | 99/2,493 (4.0)  35/126 (28.0)  31/1,051 (3.0)  33/1,316 (2.5) | 118/2,038 (5.8)  98/292 (33.6)  12/532 (2.3)  8/1214 (0.7) | 13/294 (4.4)  13/40 (32.5)  0 (0.0)  0.(0.0) | 198/814 (24.3)  52/134 (38.8)  146/149 (97.0)  0/531 (0.0) |
| **Number of oocytes retrieved**  *Study Center 1*  *Study Center 2*  *Study Center 3* | 5 [3-7]  7 [5-8]  6 [4-8] | 7 [5-8]  7 [5-8]  7 [5-8]  7 [5-8] | 6 [4-8]  5 [3-7]  7 [5-8]  6 [4-8] | 3 [2-5]  3 [1-4]  3 [1-5]  3 [2-5] | 3 [1-4]  2 [1-4]  3 [1-4]  3 [2-5] |

Values are median and 25%-75% interquartile range

Study Center 1: ANDROFERT (Brazil); Study Center 2: Anatolia IVF (Turkey); Study Center 3: My Duc Hospital (Vietnam)
