# Supplementary material for: Low Prognosis by the POSEIDON Criteria in Women Undergoing Assisted Reproductive Technology: A Multicenter and Multinational Prevalence Study of Over 13,000 Patients
Source: Front Endocrinol (Lausanne). 2021 Mar 12;12:630550. doi: 10.3389/fendo.2021.630550 (PMC8006427; doi:10.3389/fendo.2021.630550)
Supplement: Supplementary file 4 [file DataSheet_4.docx]

**Supplementary Table 4**. Demographic and treatment characteristics of total population by study center

|  | **Study Center 1**  **n=1,063** | **Study Center 2**  **n=3,635** | **Study Center 3**  **n=8,448** | **P-value** |
| --- | --- | --- | --- | --- |
| **Age (years)** | 36 [33-39] | 31 [27-35] | 33 [29-36] | <0.001^a,b^ |
| **BMI (kg/m^2^)** | 23.4 [21.5-26.0) | 24.7 [22.0-28.0] | 20.7 [19.5-22.3] | 0.02^a,b^ |
| **Infertility duration (months)** | 48 [24-96] | 48 [24-84] | 48 [24-72] | <0.001^a^ |
| **Primary indication ART**  *Male*  *Endometriosis*  *Ovulatory*  *Tubal*  *Unexplained* | 488/1,063 (45.9)  121/1,063 (11.4)  107/1,063 (10.1)  79/1063 (7.4)  268/1,063 (25.2) | 1,209/3,635 (33.2)  383/3,635 (10.5)  578/3,635 (15.9)  150/3,635 (4.1)  1,315/3,635 (36.1) | 2,796/8,448 (30.9)  538/8,448 (6.4)  1,151/8,448 (12.7)  1,439/8,448 (17.0)  2,524/8,448 (29.7) | <0.001^c^ |
| **Ovarian reserve**  *AFC (n)*  *AMH (ng/mL)* | 8 [6-13]  1.7 [0.7-3.0) | 14 [10-20]  1.8 [0.9-3.1] | 13 [3-35]  3.8 [0.3-13.4] | <0.001^a,b^  <0.001^a,b^ |
| **Duration of stimulation (days)** | 10 [10-10] | 9 [8-10] | 9 [8-10] | <0.001^a,b^ |
| **GnRH analogue (Antagonist)**  *Antagonist*  *Agonist* | 1,003/1,063 (94.3)  60/1,063 (5.7) | 1,723/3,635 (47.4)  1,912/3,635 (52.6) | 8,448/8,448 (100.0)  0/8,448 (0.0) | <0.001^c^ |
| **Total gonadotropin dose (IU)** | 2,700 [1,125-4,950] | 1,725 [1,050-4,950] | 2,700 [1,200-4,800] | <0.001^a,b^ |
| **Gonadotropin (HMG)**  *HMG*  *Rec-FSH*  *Rec-FSH + HMG*  *Rec-FSH + rec-LH* | 0/1,063 (0.0)  565/1,063 (53.1)  1/1,063 (0.1)  497/1,063 (46.8) | 257/3,635 (7.1)  2,198/3,635 (60.5)  981/3,635 (27.0)  199/3,635 (5.4) | 65/8,448 (0.8)  2,247/8,448 (26.6)  5,880/8,448 (69.6)  256/8,448 (3.0) | <0.001^c^ |
| **Trigger:**  *hCG*  *GnRH agonist* | 661/1,063 (62.2)  402/1,063 (37.8) | 3,173/3,635 (87.3)  462/3,635 (12.7) | 7,097/8,448 (84.0)  1,351/8,448 (16.0) | <0.001^c^ |
| **Number of oocytes retrieved** | 8 [5-14] | 10 [7-13] | 12 [7-17] | <0.001^a,b^ |

^a^Kruskal-Wallis test; values are median and 25%-75% interquartile range

^b^Non-parametric comparison for each pair using Wilcoxon test; values are median and 95% interquartile range. P values different than those reported in table shown below:

*AMH: Study Center (SC) 1 vs. SC 2: p=0.16;*

*Duration of stimulation: SC 1 vs. SC 3: p=0.03;*

^c^Pearson χ2 test. Values are number (percentage).
