# Supplementary material for: Low Prognosis by the POSEIDON Criteria in Women Undergoing Assisted Reproductive Technology: A Multicenter and Multinational Prevalence Study of Over 13,000 Patients
Source: Front Endocrinol (Lausanne). 2021 Mar 12;12:630550. doi: 10.3389/fendo.2021.630550 (PMC8006427; doi:10.3389/fendo.2021.630550)
Supplement: Supplementary file 5 [file DataSheet_5.docx]

**Supplementary Table 5.** Association of patient characteristics and the condition ‘POSEIDON group 3 or group 4’.

| **Term (unit)** | **Estimate** | **Std Error** | | **P value** | **Odds ratio*** | **Lower 95%** | **Upper 95%** |
| --- | --- | --- | --- | --- | --- | --- | --- |
| Intercept | 16.4226 | 0.7408 | | <0.0001 |  |  |  |
| Female age (year) | 0.4059 | 0.0162 | | <0.0001 | 1.5007 | 1.4535 | 1.5494 |
| BMI (Kg/m2) | 0.0696 | 0.0208 | | 0.0008 | 1.0293 | 1.0293 | 1.1167 |
| Infertility duration (month) | 0.0023 | 0.0010 | | 0.0223 | 1.0023 | 1.0003 | 1.0043 |
| Primary treatment indication (Female factor) | 1.3392 | 0.1135 | | <0.0001 | 6.9286^1^ | 4.7645 | 10.0757 |
| Study Center (1-3) | -0.3133 | 0.2688 | | 0.2463 | 0.6045^2^ | 0.2626 | 1.3912 |
| Study Center (2-3) | 0.1899 | 0.1719 | | 0.2728 | 1.0688^3^ | 0.7509 | 1.5213 |
| Response: POSEIDON groups 3 or 4=yes  Distribution: binomial  Estimation method: nominal logistic  Number of Parameters: 6  Whole model test: ChiSquare=1816.97; p<0.0001 | | | BIC: 2225.94  AICc: 2171.03  RSquare: 0.4574  Area under the curve ROC curve: 0.94  Lack of fit test: 1.0 | | | | |

Study Center (SC) 1: ANDROFERT (Brazil); SC2: Anatolia IVF (Turkey); SC3: My Duc Hospital (Vietnam)

*Per unit change in regressor (independent variable)

^1^Odds ratio for female factor vs. no female factor (unexplained or male factor)

^2^Odds ratio for Study Center 1 vs. Study Center 2

^3^Odds ratio for Study Center 2 vs. Study Center 3
