# Supplementary material for: Low Prognosis by the POSEIDON Criteria in Women Undergoing Assisted Reproductive Technology: A Multicenter and Multinational Prevalence Study of Over 13,000 Patients
Source: Front Endocrinol (Lausanne). 2021 Mar 12;12:630550. doi: 10.3389/fendo.2021.630550 (PMC8006427; doi:10.3389/fendo.2021.630550)
Supplement: Supplementary file 6 [file DataSheet_6.docx]

**Supplementary Table 6.** Association of patient and treatment characteristics and the condition ‘POSEIDON group 1 or group 2’.

| **Term (unit)** | **Estimate** | **Std Error** | **P value** | | **Odds ratio*** | **Lower 95%** | **Upper 95%** |
| --- | --- | --- | --- | --- | --- | --- | --- |
| Intercept | 0.8936 | 0.2951 | 0.0026 | |  |  |  |
| Female age (year) | 0.0289 | 0.0055 | <0.0001 | | 1.0293 | 1.0181 | 1.0407 |
| BMI (Kg/m2) | 0.0367 | 0.0071 | <0.0001 | | 1.0374 | 1.0229 | 1.0521 |
| Infertility duration (month) | -4.52e-5 | 0.0005 | 0.9684 | | 0.9999 | 0.9988 | 1.0010 |
| AFC (n) | -0.1727 | 0.0052 | <0.0001 | | 0.8313 | 0.8328 | 0.8500 |
| Primary treatment indication (Female factor) | 0.2429 | 0.0349 | <0.0001 | | 1.4975^1^ | 1.3399 | 1.6736 |
| Stimulation duration (day) | -0.0643 | 0.0218 | 0.0032 | | 0.4330 | 0.2480 | 0.7560 |
| Total gonadotropin dose (IU) | -8.22e-5 | 4.57e-5 | 0.0723 | | 0.5991 | 0.3427 | 1.0473 |
| GnRH antagonist protocol | 0.2518 | 0.0429 | <0.0001 | | 1.6547^2^ | 1.3983 | 1.9582 |
| Gonadotropin [HMG] | 0.3342 | 0.1285 | 0.0093 | | 2.4809^3^ | 1.7759 | 3.4659 |
| Gonadotropin  [rec-FSH+HMG] | 0.1270 | 0.0618 | 0.0401 | | 2.0165^4^ | 1.7673 | 2.3007 |
| Gonadotropin [rec-FSH] | -0.5743 | 0.0625 | <0.0001 | | 0.4030^5^ | 0.2885 | 0.5630 |
| Study Center (1-2) | -0.4768 | 0.1402 | 0.0007 | | 0.2558^6^ | 0.1664 | 0.3930 |
| Study Center (2-3) | 0.8864 | 0.0873 | <0.0001 | | 3.6545^7^ | 3.0434 | 4.3884 |
| Response: POSEIDON groups 1 or 2 =yes  Distribution: binomial  Estimation method: nominal logistic  Number of Parameters: 13  Whole model test: ChiSquare=3156.77; p<0.0001 | | | | BIC: 10697.5  AICc: 10588.8  RSquare: 0.23  Area under the curve ROC curve: 0.82  Lack of fit test: 0.09 | | | |

Study Center (SC) 1: ANDROFERT (Brazil); SC2: Anatolia IVF (Turkey); SC3: My Duc Hospital (Vietnam)

*Per unit change in regressor (independent variable)

^1^Odds ratio for female factor vs. no female factor (unexplained or male factor)

^2^Odds ratio for GnRH antagonist vs. GnRH agonist

^3^Odds ratio for gonadotropin utilized: HMG vs. rec-FSH

^4^Odds ratio for gonadotropin utilized: rec-HMG vs. rec-FSH

^5^Odds ratio for gonadotropin utilized: rec-FSH vs. HMG

^6^Odds ratio for Study Center 1 vs. Study Center 2

^7^Odds ratio for Study Center 2 vs. Study Center 3
